# Bi_3_O_4_Br nanosheets immobilized in chitosan microspheres as efficient and recyclable hybrid catalysts for water treatment

**DOI:** 10.1039/d6ra02619b

**Published:** 2026-05-22

**Authors:** Hassan Ait Yachou, Abdelmalik Brik, Mustapha El Kadiri, Taha El Assimi, Hicham Ben Youcef, Géraldine Gouhier, Jamal El Haskouri, Abdellatif El Meziane, Abdelkrim El Kadib, Mohammed Lahcini

**Affiliations:** a IMED-Lab, Faculty of Sciences and Techniques, Cadi Ayyad University Avenue Abdelkrim Elkhattabi, B.P. 549 40000 Marrakech Morocco m.lahcini@uca.ma; b High Throughput Multidisciplinary Research Laboratory (HTMR), College of Chemical Sciences and Engineering (CCSE), Mohammed VI Polytechnic University (UM6P) Lot 660 Hay Moulay Rachid Ben Guerir Morocco; c Normandie Université, COBRA, UMR6014, FR 3038, INSA Rouen, CNRS, IRCOF 76821 Mont-Saint-Aignan France; d Institut de Ciència dels Materials (ICMUV), Universitat de València Valencia 46071 Spain; e Laboratory of Agrobiotechnology and Bioengineering, Department of Biology, Cadi Ayyad University 40000 Marrakech Morocco; f Department Euromed Research Center, Engineering Division, Euro-Med University of Fes 30070 Morocco; g Chemical and Biochemical Sciences (CBS), Mohammed VI Polytechnic University Lot 660, Hay Moulay Rachid Ben Guerir 43150 Morocco

## Abstract

Access to clean water is a critical global priority. Thus, photocatalysis using semiconducting materials has emerged as a promising technology for wastewater treatment. Herein, a novel Bi_3_O_4_Br@Chitosan hybrid composite was successfully prepared by immobilizing Bi_3_O_4_Br in chitosan (CS) beads. First, Bi_3_O_4_Br was prepared *via* a solvothermal process, followed by its physical embedding in the CS matrix *via* a simple coprecipitation method. The surface morphology, elemental composition, crystal structure, and optical properties of the Bi_3_O_4_Br@CS material were comprehensively investigated using SEM, EDS, FTIR, XRD, TGA, zeta potential, Raman, and UV-vis spectroscopy, indicating excellent compatibility, multifunctional structure, and high structural robustness. Consequently, the Bi_3_O_4_Br@CS catalyst exhibits high efficiency in the UV-light-driven photodegradation of Rhodamine B (RhB), achieving 88% RhB degradation within 150 minutes and total reduction of 4-nitrophenol (4-NP) in the presence of NaBH_4_ within 5 min at room temperature. Additionally, the catalyst shows good stability and can be reused over seven successive cycles without significant loss of activity. Therefore, the combination of adsorption capacity and photocatalytic activity within this hybrid catalyst provides an efficient and practical approach for wastewater treatment applications.

## Abstract 2

Access to clean water is a critical global priority.

## Graphical abstract

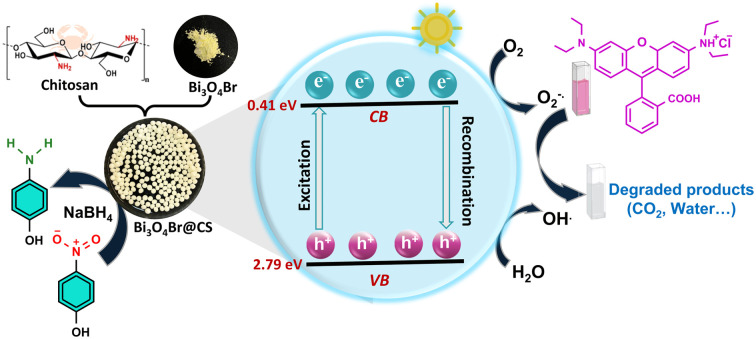

## Introduction

Water pollution and the escalating scarcity of safe drinking water are among the most critical environmental and public health challenges of the modern era.^1–3^ Driven by rapid demographic expansion and ongoing industrialization, severe overexploitation of water resources has significantly increased the concentration of recalcitrant organic contaminants, such as industrial dyes, pharmaceutical phenols, and agricultural pesticides, in global aquatic ecosystems.^4,5^ While conventional remediation strategies, including chlorination, ozonation, and advanced membrane filtration, are widely employed, their large-scale application is frequently hindered by prohibitive operational costs, stringent technical requirements, and the generation of hazardous secondary byproducts.^6^ Consequently, semiconductor inorganic–organic photocatalysis, such as 6,13-pentacenequinone/TiO_2_ (PQ/Ti)^7^ has emerged as a highly efficient, cost-effective, and environmentally benign alternative that thoroughly mineralizes pollutants rather than merely transferring them between phases.^8,9^

Thus, extensive research has focused on developing novel photocatalytic architectures for wastewater treatment, *e.g.,* metal oxides,^10^ metal sulfides,^11^ metal phosphides,^12^ metal nitride,^13^ metal carbide,^14^ perovskite-based materials,^15^ graphene-based materials,^16^ graphitic carbon nitride (g-C_3_N_4_),^17^ and metal–organic framework.^18^ Among these, metal oxides, particularly bismuth oxyhalides (BiOX),^1,2^ are particularly attractive due to their low toxicity, low cost, and important photoredox properties.^19–24^ Importantly, bismuth oxybromide (BiOBr) has particularly attracted immense attention as a superior photocatalytic material.^22,24^ This prominence is attributed to their unique layered structure, enhanced visible-light absorption, low toxicity, and exceptional charge-separation efficiency, which collectively enable robust environmental remediation applications.

To further optimize the intrinsic photocatalytic efficacy of BiOBr, researchers have actively engineered their morphological and electronic profiles.^23,25,26^ For instance, precisely modulating the pH during synthesis yields distinct morphologies that significantly affect and improve the degradation rates of organic dyes and phenolic compounds. Beyond morphological tuning, constructing heterojunctions is a primary strategy to suppress the rapid recombination of photogenerated electron–hole pairs.^27–29^ Coupling BiOBr with specialized conductive modifiers, such as carbon quantum dots, two-dimensional MXenes, and BiVO_4,_ has been shown to significantly accelerate the degradation kinetics of persistent organic pollutants.^27,30^ Recently, Niu *et al.* developed a Z-scheme Bi_3_O_4_Br/BiVO_4_ nanocomposite *via* microwave hydrothermal methods to optimize organic pollutant degradation. This heterojunction architecture suppresses carrier recombination, generating reactive radicals that completely degrade methyl orange in 80 minutes under sunlight. The material also removes 85% of norfloxacin in 75 minutes while maintaining high structural stability across multiple cycles. Its efficiency and eco-friendly nature suggest significant potential for sustainable, industrial-scale wastewater purification.^30^ These advanced materials demonstrate remarkable efficiency in the degradation of toxic organic dyes under visible light. By utilizing a richer bismuth content, they facilitate more effective charge separation and enhanced light absorption compared to standard alternatives.^1,23,25,26,31,32^

Despite these catalytic advantages, the practical, industrial-scale deployment of powdered BiOBr is constrained by significant logistical challenges in post-treatment catalyst recovery and physical recyclability. To resolve this limitation, immobilizing the active photocatalytic powder onto stable macroscopic support structures has proven highly effective and challenging.^30,33^ For instance, natural biopolymers, especially polysaccharides, represent ideal candidates to meet this requirement due to their multi-functional structure, biocompatibility, and diverse availability, which increase their affinity to several organic pollutants.^34–38^ Among them, chitosan (CS), provides more flexibility in catalysis due to the presence of functional groups such as amino (–NH_2_) and hydroxyl (–OH) functionalities that exhibit a strong inherent chemical affinity for both the catalytic species and the target aqueous pollutants, thereby establishing a highly stable, reusable, and sustainable platform for advanced water purification.^2,19,39–41^ Consequently, integrating the distinct photocatalytic proficiency of BiOBr with the exceptional adsorption capacity and structural flexibility of CS presents a highly promising research frontier. This synergistic combination is anticipated to yield novel hybrid composites characterized by unique catalytic properties and enhanced operational robustness, providing a strong motivation for continued innovation in sustainable wastewater remediation.^2,3,27^

Despite these important advances, current BiOX–chitosan and Bi_3_O_4_Br-based systems still face several limitations, including limited structural integration, difficulties in catalyst recovery, and the lack of multifunctional systems combining different catalytic processes. In many cases, the materials remain in powder form, which restricts their practical use in real water treatment conditions. Although Bi_3_O_4_Br offers improved light absorption and charge separation compared to BiOBr, its integration into a stable, recoverable, and multifunctional architecture remains insufficiently explored. Herein, we bridge this gap by immobilizing Bi_3_O_4_Br nanosheets within chitosan beads *via* a facile synthetic strategy, creating a robust platform that prevents nanoparticle leaching and ensures seamless catalyst recovery. This approach allows us to combine the advantages of nanoscale catalytic activity with a macroscopic structure that facilitates recovery and reuse. In addition, the system exhibits a dual functionality, enabling both RhB photodegradation and 4-nitrophenol reduction within the same material, which represents a clear improvement over previously reported system.

## Experimental section

### Starting materials and reagents

Chitosan (CS) with a molecular weight of 600 000 kD was obtained from Sigma-Aldrich. Its deacetylation degree was calculated using pH metric titration of about (∼95%). All other starting materials, solvents, and reagents, including bismuth nitrate pentahydrate (Bi(NO_3_)_3_·5H_2_O), sodium bromide (NaBr), sodium hydroxide (NaOH), acetic acid (CH_3_COOH), and RhB, are commercially available and used as received.

### Characterization methods

The composition and crystal phase structure of the prepared hybrid materials were investigated by XRD measurement using a Rigaku X-ray diffractometer equipped with a Cu-Kα X-ray source with a wavelength of (*λ* = 1.5418 Å). The XRD patterns were recorded in a 2*θ* range of 5–90° at step size of 0.02°/step and a scanning speed of 2–5° min^−1^. The surface morphology and microstructure of the prepared hybrid materials were observed using SEM. SEM observations were performed using “Tescan Vega3” microscope, equipped with an energy dispersive X-ray spectrometry (EDS) analyser, allowing the determination of chemical compositions. The preparation of the samples consists in depositing the materials as powder or pellets on an aluminum sample holder with a double-sided carbon adhesive. TGA was used to investigate the thermal behavior and stability of the obtained material. TGA was carried out using a 0650-0580 (192.168.1.2) TGA analyzer in a temperature range of 10–700 °C with a heating rate of 10 °C min^−1^. The chemical structure of the prepared materials was further analyzed by FTIR spectroscopy analysis. The FTIR spectra were recorded on a PerkinElmer spectrum 100 FTIR spectrophotometer. The samples were grinded with KBr powder as a reference matrix (standard) and then pressed to form transparent pellets in IR radiation. The optical properties and the light absorption ability of Bi_3_O_4_Br sheets were studied by UV-visible diffuse reflectance spectroscopy recorded on a Shimadzu (UV-2600) spectrophotometer equipped with an integrating sphere in a wavelength range of 200–1200 nm. The measurements were carried out in a transparent quartz cuvette with an optical path of 1 cm. For this, the sample was prepared by the dispersion of Bi_3_O_4_Br sheets in water with concentration of 1 mg mL^−1^. The chemical structure of the Bi_3_O_4_Br sheets were also investigated by Raman spectroscopy analysis using a NanoSp confotec-MR520. The samples were analyzed using a “LCM-S-111” laser with a wavelength of 532 nm in a 1200 line per mm network. Particle size distribution and zeta potential were measured in triplicate at 25 °C using a Malvern ZetaSizer instrument to determine the particle size distribution and zeta potential of the materials. For this, the sample was prepared by dispersing Bi_3_O_4_Br in water with a concentration of 2.5 mg mL^−1^, and charged in the measurement cell, which was a “disposable folded capillary cell (DTS1070)”.

### Catalysts preparation and utilization

#### Synthesis of Bi_3_O_4_Br sheets

Bi_3_O_4_Br sheets were prepared through a solvothermal synthesis in a water/ethylene glycol mixture using Bi(NO_3_)_3_·5H_2_O and NaBr as starting materials, followed by thermal treatment at 500 °C. To achieve this, 3 g of Bi(NO_3_)_3_·5H_2_O (∼6.18 mmol) were mixed with 80 mL of a mixture of water/ethylene glycol (1 : 1, v/v), and sonicated for about 10 min. Then, 0.5 equiv. of NaBr (317.9 mg, 3.09 mmol) was added to the above mixture under magnetic stirring. After about 15 min, the mixture was transferred to a 100 mL Autoclave and heated at 180 °C for 17.5 h. Next, the precipitates were collected by centrifugation, washed several times with distilled water and methanol, and oven-dried in air at ∼75 °C. Finally, the resulting product was calcined at 500 °C for 2 hours with a heating rate of about 5 °C min^−1^ (Fig. S1, SI).

#### Preparation of Bi_3_O_4_Br@CS beads

Bi_3_O_4_Br@CS hybrid beads were obtained through a co-precipitation method. Briefly, 400 mg of CS and 150 mg of Bi_3_O_4_Br were sonically dispersed in 20 mL of H_2_O for 1 h. Then, 200 µL of acetic acid (1% v/v) was added to the above solution under magnetic stirring to dissolve the CS (pH ∼3). The formation of CS beads was achieved by instantaneous precipitation *via* dropping the obtained solution into 200 mL of NaOH (2 M). After 1 hour in the alkaline solution, the Bi_3_O_4_Br@CS beads were filtered and washed with distilled water several times until a neutral pH solution (Fig. S2, SI). The choice of a chitosan content of 25 wt% was guided by both practical and functional considerations. During preliminary tests, lower chitosan contents resulted in fragile beads that were difficult to handle, while higher contents tended to partially block the active sites of Bi_3_O_4_Br, limiting catalytic accessibility. Therefore, this composition was selected as a reasonable balance between mechanical stability, porosity, and catalytic efficiency, in agreement with previous studies on chitosan-based hybrid systems.

#### Adsorption/desorption study

Adsorption/desorption ability is a crucial parameter in the heterogeneous catalytic process. This feature is highly beneficial for photodegradation and reduction reactions; thus, in control experiments, the adsorption/desorption of 6 mL of aqueous RhB (5 mg L^−1^) and 4-NP (10 mg L^−1^) solution over pure CS beads and Bi_3_O_4_Br@CS composites were investigated in the dark, under UV light irradiation, and under ambient conditions. At a regular time interval, aliquots of both RhB and 4-NP were collected from the reaction media and analyzed using UV-vis.

#### Photodegradation of RhB

The photocatalytic behavior of the Bi_3_O_4_Br@CS hybrid materials was investigated in the photodegradation of organic dyes under UV light irradiation. For this, RhB was chosen as a model organic dye. The photodegradation experiments of RhB over Bi_3_O_4_Br@CS hybrid materials were carried out in a quartz cuvette (25 mL) covered with a Teflon cover. In a typically, the quartz cuvette was charged with a certain amount of Bi_3_O_4_Br@CB and 6 mL of RhB (5 mg L^−1^) aqueous solution with pH ∼6.6. The reaction was stirred under simulated UV light using a Philips HPL-N 125 W lamp with a wavelength of 365 nm as a light source. The progress of the photocatalytic reaction was performed by withdrawing a aliquot of RhB solution at regular intervals and monitoring the absorbance *vs.* wavelength spectra using the UV-vis.

#### Reduction of 4-NP to 4-aminophenol

The catalytic performance of Bi_3_O_4_Br@CS hybrid beads in the reduction of 4-NP was performed in an aqueous solution using NaBH_4_ as a reducing agent. In a typical reduction process, a mixture of 6 mL of 4-NP aqueous solution (10 mg L^−1^), Bi_3_O_4_Br@CS and 2 mL NaBH_4_ (0.5, 1 or 2 M) was stirred at room temperature at selected period of time. The progress of the reduction process was determined by monitoring the absorbance band of 4-NP at *λ*_max_ = 402 nm using UV-vis spectrophotometer.

## Results and discussion

### Catalysts characterization

**Fig. 1:**
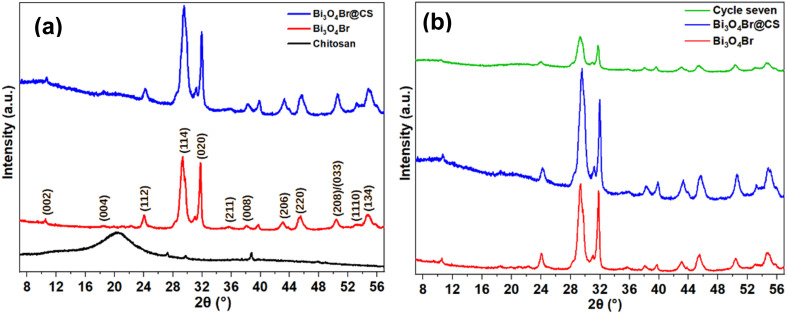
XRD patterns (a) of pure CS, Bi_3_O_4_Br sheets, and (b) Bi_3_O_4_Br@CS before and after 7 photodegradation cycles.

Highly crystalline Bi_3_O_4_Br nanosheets were successfully obtained through a solvothermal route followed by calcination. As observed in SEM and TEM images (Fig. 3), the material exhibits a lamellar morphology, which can enhance surface exposure and facilitate charge separation. This structural feature is consistent with the good photocatalytic performance observed in our system. To overcome the inherent recovery challenges of powdered suspensions, these nanosheets were successfully immobilized within macroscopic CS microspheres using a rapid alkaline co-precipitation strategy. The resulting Bi_3_O_4_Br@CS hybrid composite directly integrates the robust physical adsorption capacity of the biopolymer network with the potent photo-oxidation capabilities of the semiconductor. This synergistic coupling provides a stable, easily recoverable, and highly efficient dual-functional platform for advanced wastewater remediation. XRD analysis indicates that Bi_3_O_4_Br diffractogram, show the presence of peaks at 10.56°, 18.52°, 24°, 29.39°, 31.80°, 35.70°, 38.10°, 43.13°, 45.23°, 50.44° and 54.53° were respectively indexed to (002), (004), (112), (114), (020), (211), (008), (206), (220), (208)/(033), (110) and (134) diffraction planes confirm the successful preparation of pure and crystallin orthorhombic Bi_3_O_4_Br (Fig. 1), exhibiting high thermal stability as confirmed by TGA and crystal size of about 345.5 Å as calculated using the Debye Scherrer formula (Fig. S3, SI).^23,25,27,42–44^ For pure CS patterns, the peaks at 2*θ* values of about 12.56° and 20.16° are well indexed to the (020) and (101) reflection planes of the amorphous phase of CS, respectively.^45^ After the combination, the Bi_3_O_4_Br@CS composite shows a relatively similar profile to Bi_3_O_4_Br, this observation is consistent with the TGA observation (Fig. S3, SI), which confirms the successful process. Nevertheless, after incorporating the Bi_3_O_4_Br into the CS structure, the diffraction peaks characteristic of the amorphous phase of CS were clearly decreased, indicating the high crystallinity of the obtained materials. Moreover, XRD patterns of the reused materials clearly confirmed that no noticeable changes were observed even after 7 photocatalytic runs (Fig. 1).

**Fig. 2:**
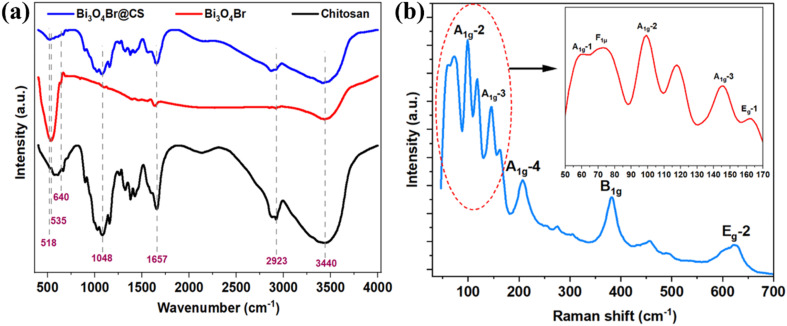
(a) FTIR spectra of pure chitosan, Bi_3_O_4_Br sheets, and Bi_3_O_4_Br @CS, (b) Raman spectra of Bi_3_O_4_Br sheets.

While FTIR analysis of pure CS reveals a strong and broad absorbent band between 3000–3700 cm^−1^ ascribed to the stretching vibration of –OH and –NH_2_ groups. Similarly, the absorption band at 2800–3000 cm^−1^ is attributed to C–H (sp^3^) groups. The absorption band at 1650 cm^−1^ is assigned as C=O groups (amide I) vibrations. The absorption band at 1427 cm^−1^ is due to C–H wagging. The absorption bands at 1375 cm^−1^ and 1079 cm^−1^ are assigned to the stretching vibration of C–N and C–O groups, respectively. Finally, the absorption band at 598 cm^−1^ is due to N–H stretching vibration. For the Bi_3_O_4_Br sheets, the absorption bands at 1638 and 3440 cm^−1^ could be attributed to the bending vibrations of O–H bond related to the adsorbed water molecules.^31^ The absorption band at 535 cm^−1^ could be ascribed to the stretching mode of the Bi–O bond of Bi_3_O_4_Br^31^ as further confirmed by Raman analysis. Additionally, the FTIR spectra of Bi_3_O_4_Br@CS hybrid materials showed much similar absorbent bands of pure CS, the major differences are the shift of the broad band observed between 535–640 cm^−1^ to the lower wavenumber, which could be due to the strong interaction between the amino groups –NH_2_ and Bi_3_O_4_Br sheets (Fig. 2a). Moreover, Raman analysis is consistent with FTIR observation, displays various vibration modes for Bi_3_O_4_Br sheets, shifts at 60.82, 99.35, and 162.3 cm^−1^ could respectively be assigned to the A_1g_ external, A_1g_ internal, and E_g_ internal of Bi–Br stretching modes.^25^ The band at 73.68 cm^−1^ corresponds to the F_1µ_ stretching mode of the O–Bi–O bond.^25^ The A_1g_ and E_g_ modes of Bi–O covalent bonds are situated at 206.8 and 621.94 cm^−1^, respectively.^25^ The two modes at 145.5 and 382.56 cm^−1^ belong to the A_1g_-3 of B–O vibration mode and B_1g_ mode caused by the movement of oxygen atoms (Fig. 2b).^25^

**Fig. 3:**
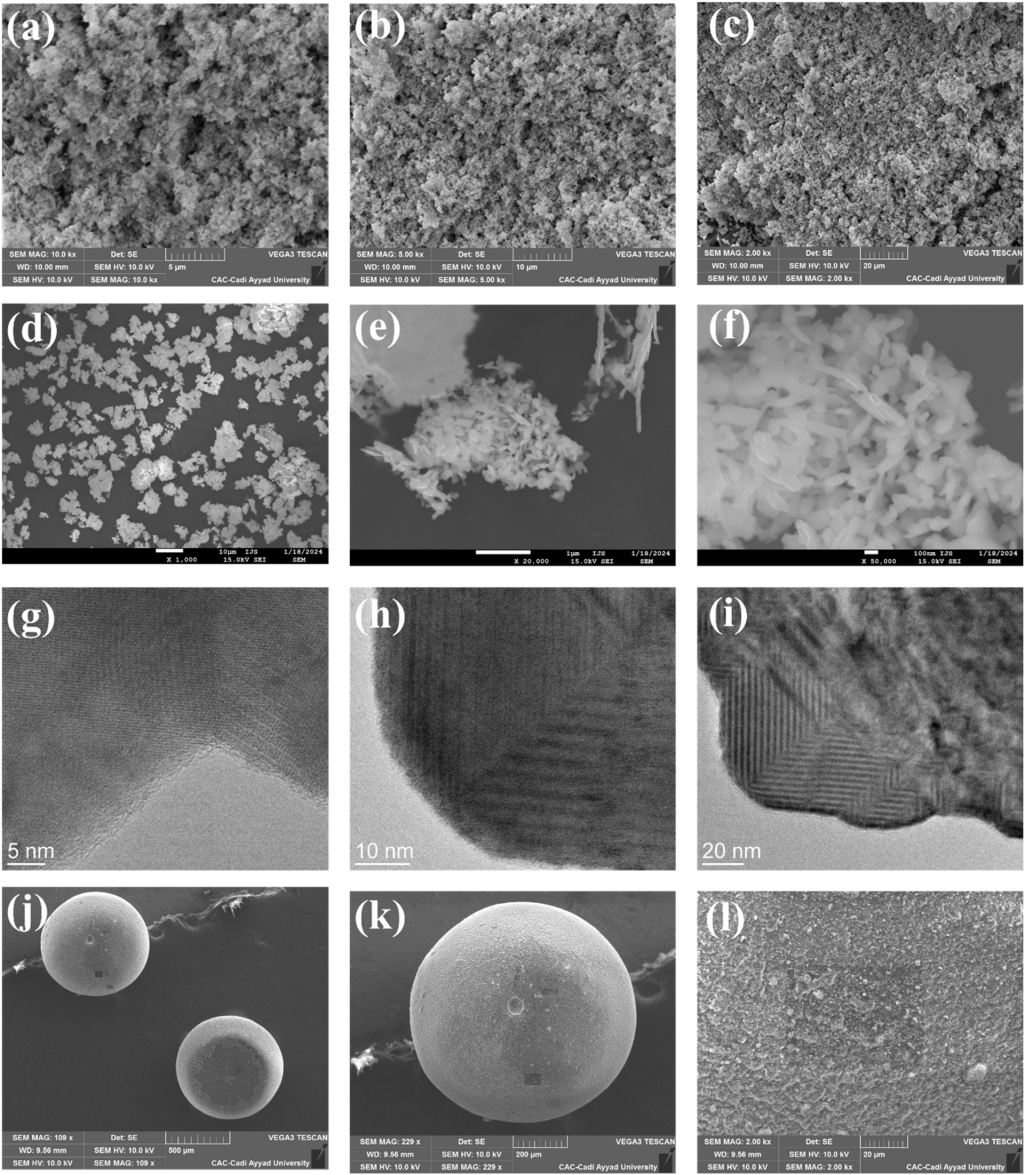
(a–c) SEM, (d–f) FE-SEM, and (g–i) TEM images of Bi_3_O_4_Br sheets; (j–l) SEM images of Bi_3_O_4_Br@CS hybrid materials.

**Fig. 4:**
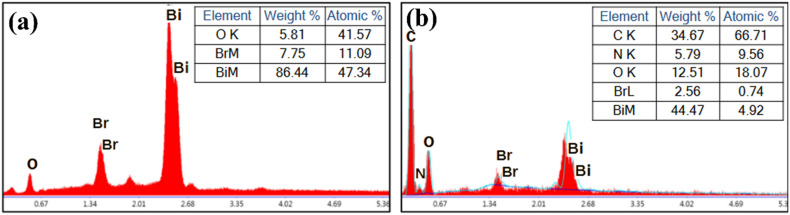
EDS spectra: (a) Bi_3_O_4_Br, (b) Bi_3_O_4_Br@CS.

Additionally, SEM was used to study the morphology of the prepared samples. The SEM, FE-SEM, and TEM images of Bi_3_O_4_Br sheets and Bi_3_O_4_Br@CS hybrid materials are shown in Fig. 3. SEM/FE-SEM images (Fig. 3a–f) show that the prepared Bi_3_O_4_Br particles are ultrathin sheets, uniform in shape, and exhibit a porous network, which was further confirmed by TEM as shown in the images (Fig. 3g–i). Furthermore, SEM images (Fig. 3j–l) revealed that Bi_3_O_4_Br@CS beads were spherical in shape with a uniform dispersion of Bi_3_O_4_Br particles on their surface. In addition, EDS has been used to give further information about the elemental composition and chemical structure of the prepared materials (Fig. 4). Likewise, the EDS spectra of Bi_3_O_4_Br sheets (Fig. 4a) revealed the presence of elemental bismuth (Bi), oxygen (O), and bromine (Br) in this material. After the combination, the EDS spectra of Bi_3_O_4_Br@CS hybrid materials (Fig. 4b) show the presence of Bi, Br, C, O, and N in the materials, which remain slightly similar even after three catalytic uses. These results give a clear indication of the successful incorporation and effective immobilization of Bi_3_O_4_Br in the CS structure.

**Fig. 5:**
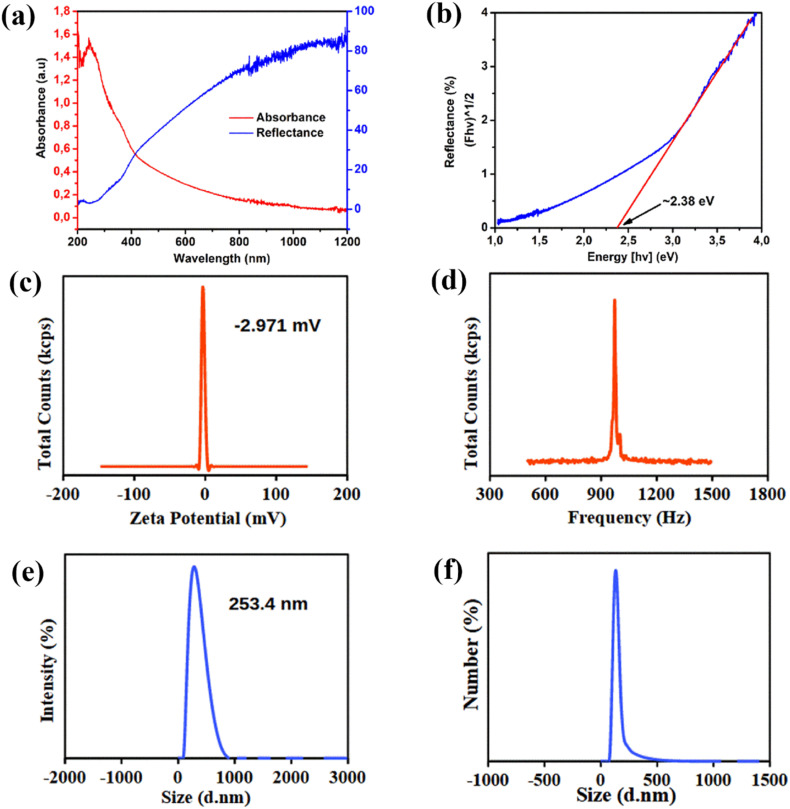
(a) UV-vis spectra (b) Tauc plot of the transformed Kubelka–Munk function [*F*(*R*)*hv*]^1/2^ against photon energy (*hv*), plots of (c) zeta potentials distribution and (d) frequency distribution, (e) size distribution by intensity and (f) size distribution by number of Bi_3_O_4_Br sheets.

Importantly, the light absorption capabilities of Bi_3_O_4_Br sheets were studied using UV-visible diffuse reflectance spectroscopy (UV-DRS). It is clearly observed that Bi_3_O_4_Br sheets display strong absorption in the UV light region (Fig. 5a). From the UV-DRS spectrum and by applying the Kubelka–Munk function (eqn (1)), the band gap value of the Bi_3_O_4_Br sheets can be estimated. This is achieved using Tauc plot of the transformed Kubelka–Munk function ([*F*(*R*)*hv*]^*n*^ = *A*(*hv* − *E*_g_)) plotted against the incident photon energy (*hv*), which is calculated from the reflectance *versus* wavelength spectra (Fig. 5b).^46–48^ As a result, the estimated band gap is approximately 2.38 eV. Furthermore, the conduction band edge potential (*E*_cb_) and valence band edge potential (*E*_vb_) of Bi_3_O_4_Br sheets can be calculated using Mulliken electronegativity theory (eqn (2) and (3)). Here, *E*_g_ is the bandgap energy of Bi_3_O_4_Br, *E*_c_ is the energy of the free electron on the hydrogen scale (*E*_c_ = 4.5 eV), and *χ* is the absolute electronegativity of Bi_3_O_4_Br. The absolute electronegativity (*χ*) of Bi_3_O_4_Br can be simply calculated according to Mulliken's definition (eqn (4)), using the constituent atoms (Bi = 4.69 eV, O = 7.54 eV, and Br = 5.64 eV), resulting in *χ* = 6.1 eV.^49,50^ Therefore, the calculated *E*_cb_ and *E*_vb_ are 0.41 eV and 2.79 eV, respectively.

1 *F*(*R*) = *K*/*S* = [(1 − *R*)^2^]/2*R*

2 *E*_cb_ = *χ* − *E*_c_ − 0.5 *E*_g_

3 *E*_vb_ = *E*_cb_ + *E*_g_

4 *χ*_Bi_3_O_4_Br_=(*χ*_Bi_^3^ × *χ*_O_^4^ × *χ*_Br_)^1/8^

In photodegradation processes, the particle size and surface charge distribution are two important factors that may determine the overall performance of any photocatalytic material. Fig. 5 illustrates the size distribution and zeta-potential spectra of the prepared Bi_3_O_4_Br sheets. The spectrum of size distribution revealed that the obtained Bi_3_O_4_Br sheets have one population with an average size value of 253.4 nm, which is consistent with TEM observation. In addition, the zeta-potential of the particles was found to be approximately −2.917 mV. These results indicate that the Bi_3_O_4_Br sheets exhibit a negative surface charge that may enhance the repulsion between the particles and prevent their agglomeration and increase the interaction with polycationic CS and RhB. Thus, contributing to the high dispersion of Bi_3_O_4_Br sheets and their stabilization within CS matrix.

### Catalytic study

**Fig. 6:**
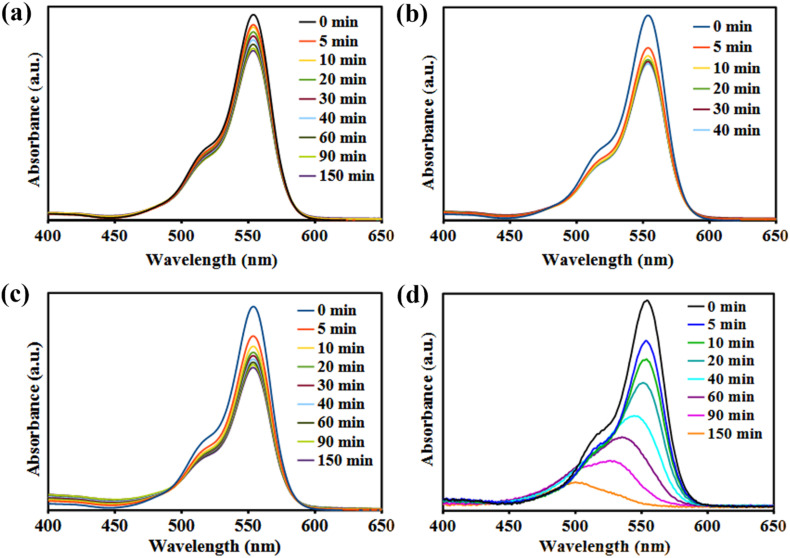
UV spectra of (a) RhB aqueous solution under UV light (b) in the presence of pure CS beads under UV light (c) in the presence of Bi_3_O_4_Br@CS in dark, and (d) in the presence of Bi_3_O_4_Br@CS under UV light.

It is widely recognized that the adsorption–desorption behavior of photocatalytic materials plays a critical role in the photodegradation of organic dyes. Consequently, the adsorption of RhB onto the Bi_3_O_4_Br@CS composite was investigated through a control experiment conducted in the dark. As illustrated in the UV-vis spectra (Fig. 6C), the Bi_3_O_4_Br@CS catalyst exhibits relatively limited adsorption capacity. This behavior is attributed to the functional groups present in both Bi_3_O_4_Br and CS; notably, the adsorption profile of the composite closely resembles that of native CS (Fig. 6B), confirming a similar interaction mechanism. To evaluate the photostability of the dye, RhB was irradiated under UV-vis light in the absence of a catalyst. No significant degradation was observed even after 150 minutes (Fig. 6A), confirming that the catalyst is essential for the degradation process. In contrast, the introduction of the Bi_3_O_4_Br@CS catalyst resulted in approximately 88% degradation of RhB within 150 minutes (Fig. 6D). These results demonstrate that the prepared catalyst exhibits high photocatalytic activity, which can be attributed to the combined effect of adsorption and photocatalysis within the hybrid system.

**Fig. 7:**
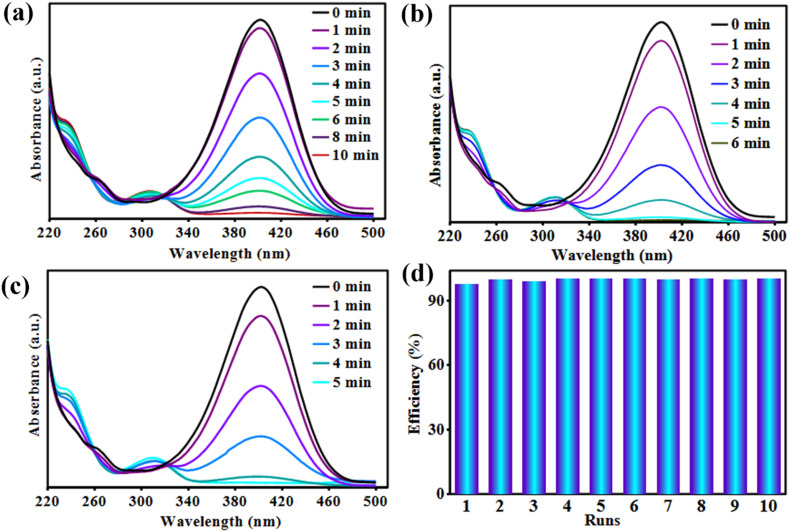
UV spectra of 4-NP aqueous aliquot withdrawn from reaction in the presence of Bi_3_O_4_Br@CS (0.4 g L^−1^) and (a) NaBH_4_ (0.5 M), (b) NaBH_4_ (1 M), (c) NaBH_4_ (2 M). (d) Recyclability graph of Bi_3_O_4_Br@CS in the reduction of 4-NP.

Similarly, the adsorption of 4-NP onto pure CS beads and the Bi_3_O_4_Br@CS composite showed no measurable change in the characteristic absorption band of 4-NP. This indicates that the 4-NP concentration remained constant, suggesting the absence of a significant adsorption–desorption process on the catalyst surface (Fig. 9). In control experiments, no reduction of 4-NP was observed when using native CS beads, even in the presence of a large excess of NaBH_4_. This confirms that the Bi_3_O_4_Br component is essential for catalytic reaction. Furthermore, no reaction occurred when using the Bi_3_O_4_Br@CS composite without NaBH_4_, nor did it occur with NaBH_4_ in the absence of the catalyst. These observations confirm that both the Bi_3_O_4_Br@CS catalyst and the NaBH_4_ reducing agent are indispensable for the process. Increasing the NaBH_4_ concentration was found to slightly enhance the reaction rate, indicating its influence on the kinetics of the reduction (Fig. 7a–c). Additionally, the Bi_3_O_4_Br@CS catalyst exhibited high efficiency and excellent reusability, maintaining stable performance over 10 consecutive cycles without significant loss of activity (Fig. 7d).

### Kinetic studies

**Fig. 8:**
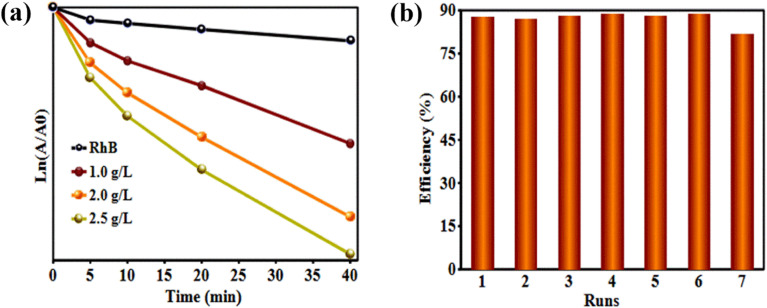
Kinetic study plots of *A*_0_/*A* and ln(*A*/*A*_0_) *versus* photodegradation time of RhB (a). Recyclability graph of Bi_3_O_4_Br@CS in the degradation of RhB (b).

The photocatalytic activity of the prepared Bi_3_O_4_Br@CS hybrid materials was evaluated in the photodegradation of RhB under UV irradiation. Control experiments were performed in the absence of a catalyst and with pure CS beads to account for potential self-photolysis and the individual contribution of the CS support. The degradation process was monitored using UV-vis spectroscopy. The time-dependent UV-vis absorption spectra for the degradation of RhB over the Bi_3_O_4_Br@CS composite are presented in Fig. 6d. As illustrated, approximately 88% of the RhB was removed within 150 minutes of UV exposure. Analysis of the kinetic curves at the 40 minute mark revealed that the degradation rate increased proportionally with the photocatalyst loading (Fig. 8b). This enhancement in performance is attributed to the increased density of active sites available within the reaction medium, which facilitates more efficient interaction between the catalyst and the organic dye molecules. Additionally, the prepared material demonstrates good recyclability in the photodegradation of RhB over 7 consecutive cycles with minimal decrease in the catalytic performance (Fig. 8b).

**Fig. 9:**
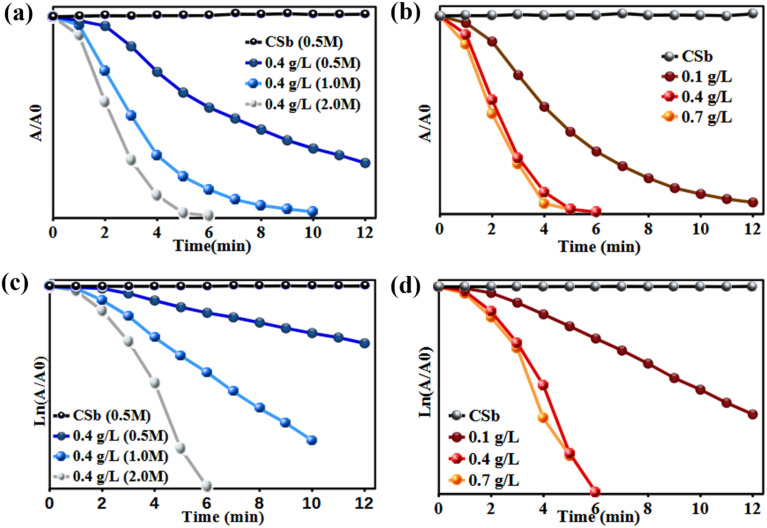
Kinetic study plots of *A*/*A*_0_ and ln(*A*/*A*_0_) *versus* reduction time of 4-NP: effect of NaBH_4_ concentration (a and c) and Bi_3_O_4_Br@CS catalyst loading (b and d).

In the same way, the UV spectra of 4-NP reduction in the presence of NaBH_4_ as a reducing agent are described in Fig. 9. It is obviously observed that in the presence of pure CS beads without Bi_3_O_4_Br@CS, the concentration of 4-NP remains constant during the reduction process. In contrast, when Bi_3_O_4_Br@CS was used as a catalyst instead of pure CS beads, the concentration of 4-NP decreased progressively with reaction time. In addition, the presence of a large excess of NaBH_4_ without a catalyst, no reduction was observed. These results indicated that the reduction process could not be achieved without Bi_3_O_4_Br@CS hybrid materials and NaBH_4_ as a hydrogen source. Moreover, it was also observed that the reduction rate increases linearly with increasing the Bi_3_O_4_Br@CS loading (Fig. 9b and c). Additionally, the effect of NaBH_4_ concentration on the 4-NP reduction was also studied in the range of (0.5, 1, and 2 M) with a fixed catalyst amount. As shown in Fig. 9a and d, it can be clearly observed that the NaBH_4_ concentration has a significant effect on the reduction rate of 4-NP. These results demonstrated that there is a linear relationship between the reduction rate and the NaBH_4_ concentration.

Recycling experiments were performed to comprehend the behavior and the stability of Bi_3_O_4_Br@CS hybrid materials in the reduction and photodegradation process. At the end of each reaction, the Bi_3_O_4_Br@CS catalyst was recovered by simple filtration, washed three times with distilled water, and then reused in another catalytic cycle. Fig. 7d and 8b show the graph obtained for both 4-NP reduction and RhB photodegradation process. It is clearly observed that the Bi_3_O_4_Br@CS hybrid materials have excellent recyclability in the reduction of 4-NP and RhB photodegradation. These materials could be reused about 7 times in the photodegradation process and 10 times in the reduction reaction with no significant loss in their catalytic performances. Consistently, our system exhibits excellent activity and good robustness, making it an ideal catalyst for water treatment.

### Proposed photodegradation and reduction mechanisms

**Fig. 10:**
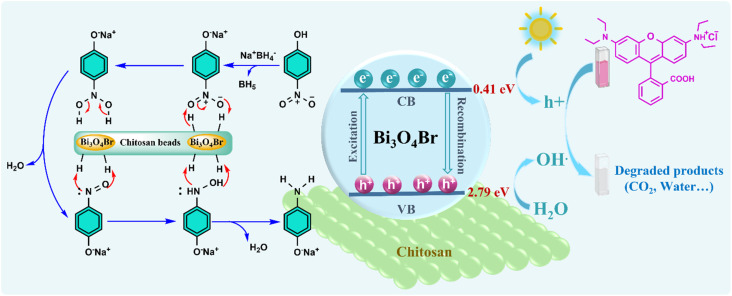
Schematic illustration of the proposed RhB photodegradation and 4-NP reduction mechanism over Bi_3_O_4_Br@CS hybrid materials.

The photodegradation reaction usually starts with the photoexcitation of Bi_3_O_4_Br by light irradiation. This process leads to the creation of electrons and holes (charge carriers) at the valence band (VB) and conduction band (CB) of the photocatalyst, respectively (eqn (5)). Then, the generated electrons and holes can participate in several photoreduction and photooxidation reactions at the surface of Bi_3_O_4_Br, respectively.^51,52^ Based on the calculated valence band position (2.79 eV), which is more positive than the oxidation potential of H_2_O/˙OH (+2.27 eV), the formation of hydroxyl radicals (˙OH) is thermodynamically feasible. In parallel, the conduction band position (0.41 eV) allows the reduction of dissolved oxygen into superoxide species (˙O_2_^−^), which are known to play a key role in dye degradation processes. In addition, the photogenerated electrons may react with dissolved oxygen to form superoxide radicals (˙O_2_^−^), which can also contribute to the degradation process (eqn (7)–(9)). Furthermore, the catalytic reduction of 4-NP was scrutinized using NaBH_4_ as a reducing agent in the presence of the Bi_3_O_4_Br@CS hybrid catalyst. The process follows a Langmuir–Hinshelwood (LH) mechanism, wherein the CS matrix plays a pivotal role by serving as a high-capacity adsorbent that concentrates 4-NP and NaBH_4_ in proximity through its abundant functional groups. This biopolymer framework ensures the uniform dispersion of Bi_3_O_4_Br active sites and prevents their aggregation, which is essential for facilitating efficient electron transfer from the NaBH_4_ donor to the 4-NP acceptor. Following the initial deprotonation of 4-NP to 4-nitrophenolate under the alkaline conditions provided by the reductant, the process proceeds *via* sequential hydrogen transfers. This pathway generates critical intermediates, including 4-nitrosophenolate and 4-hydroxylaminophenol, before reaching the final product, 4-aminophenolate. Beyond its kinetic contributions, the integration of the CS matrix enhances the structural stability of the hybrid system, enabling facile catalyst recovery and sustained catalytic activity over multiple operational cycles (Fig. 10).

5 Bi_3_O_4_Br + *hv* → e^−^ + H^+^

6 HO^−^/H_2_O + h^+^ → HO˙

7 O_2_ + h^+^ → ˙O_2_^−^

8 RhB + OH˙ → degraded products

9 RhB + H^+^ → degraded products

## Conclusion

In conclusion, a high-performance Bi_3_O_4_Br@CS hybrid photocatalyst was successfully prepared through a simple steps strategy. The composite integrates the photocatalytic properties of Bi_3_O_4_Br with the biodegradable and mechanically robust characteristics of CS, leading to a structured material in which Bi_3_O_4_Br nanosheets are uniformly embedded within chitosan microspheres. This design facilitates catalyst handling and recovery compared to conventional powder systems.

The hybrid material demonstrated effective performance in both the photodegradation of RhB under UV irradiation and the reduction of 4-NP in the presence of NaBH_4_. The catalytic activity was influenced by operational parameters such as catalyst amount and reducing agent concentration, confirming good accessibility of active sites. In addition, the composite maintained consistent performance over repeated cycles, indicating satisfactory stability and reusability.

Overall, the results show that integrating Bi_3_O_4_Br nanosheets within a chitosan matrix is an effective strategy to improve catalyst usability, particularly in terms of recovery, stability, and multifunctional performance. This approach provides a practical and sustainable pathway for designing recoverable catalytic systems with potential application in wastewater treatment.

## Conflicts of interest

The authors have no conflicts of interest to declare. All co-authors have seen and agree with the contents of the manuscript, and there is no financial interest to report. We certify that the submission is original work and is not under review at any other publication.

## Supplementary Material

RA-016-D6RA02619B-s001

## Data Availability

All data supporting this study are included in the article and its supplementary information (SI). Supplementary information is available. See DOI: https://doi.org/10.1039/d6ra02619b.
