# Supplementary material for: Bi3O4Br nanosheets immobilized in chitosan microspheres as efficient and recyclable hybrid catalysts for water treatment
Source: RSC Adv. 2026 May 22;16(30):27621–33. doi: 10.1039/d6ra02619b (PMC13202528; doi:10.1039/d6ra02619b)
Supplement: RA-016-D6RA02619B-s001 [file RA-016-D6RA02619B-s001.pdf]

Supplementary Information (SI) for RSC Advances.
This journal is © The Royal Society of Chemistry 2026

Supporting Informations

Bi_3O_4Br Nanosheets Immobilized in Chitosan Microspheres as Efficient and Recyclable Hybrid Catalysts for Water Treatment

Hassan Ait yachou [a], Abdelmalik Brik [a,b], Mustapha El Kadiri [a], Taha El Assimi [a], Hicham Ben Youcef [b], Géraldine Gouhier [c], Jamal El Haskouri[d], Abdellatif El meziane [e], Abdelkrim El Kadib [f], and Mohammed Lahcini [a,g] *

[a]IMED-Lab, Faculty of Sciences and Techniques, Cadi Ayyad University, Avenue Abdelkrim Elkhattabi, B.P. 549, 40000 Marrakech, Morocco.

[b]High Throughput Multidisciplinary Research Laboratory (HTMR), College of Chemical Sciences and Engineering (CCSE), Mohammed VI Polytechnic University (UM6P), Lot 660 Hay Moulay Rachid, Ben Guerir, Morocco.

[c]Normandie Université, COBRA, UMR6014, FR 3038, INSA Rouen, CNRS, IRCOF, 76821 Mont-Saint-Aignan, France

[d] Institut de Ci`encia dels Materials (ICMUV), Universitat de Val`encia, 46071, Valencia, Spain

[e]Laboratory of Agrobiotechnology and Bioengineering, Department of Biology, Cadi Ayyad University, 40000 Marrakech, Morocco

[f]Department Euromed Research Center, Engineering Division, Euro-Med University of Fes, 30070, Morocco

[g] Chemical and Biochemical Sciences (CBS), Mohammed VI Polytechnic University, Lot 660, Hay Moulay Rachid, Ben Guerir 43150, Morocco.

Corresponding author: Mohammed Lahcini email: m.lahcini@uca.ma

Experimental details

- **Preparation of Bi3O_4Br nanosheets**

Bi_3O_4Br nanosheets were prepared through a solvothermal synthesis in water/ethylene glycol mixture using $Bi(NO_3)_3{\cdot}5H_2O$ and NaBr as starting materials followed by thermal treatment at 500°C. To achieve this, 3 g of $Bi(NO_3)_3.5H_2O$ (~6.18 mmol) were mixed with a mixture of water (40 mL) and ethylene glycol (40 mL) and sonicated for about 10 min. Then, 317.9 mg of NaBr (~3.09 mmol) was added to the above mixture under magnetic stirring. After about 15 min, the mixture was transferred into an Autoclave of 100 mL and heated at 180°C for 17.5h. Next, the precipitates were collected by centrifugation, washed several times with distilled water and methanol and oven-dried in air at ~75°C. Finally, the resulting product was calcined at 500°C for 2 hours with a heating rate of about 5°C/min. A schematic illustration of the synthetic process is described in Figure S1.

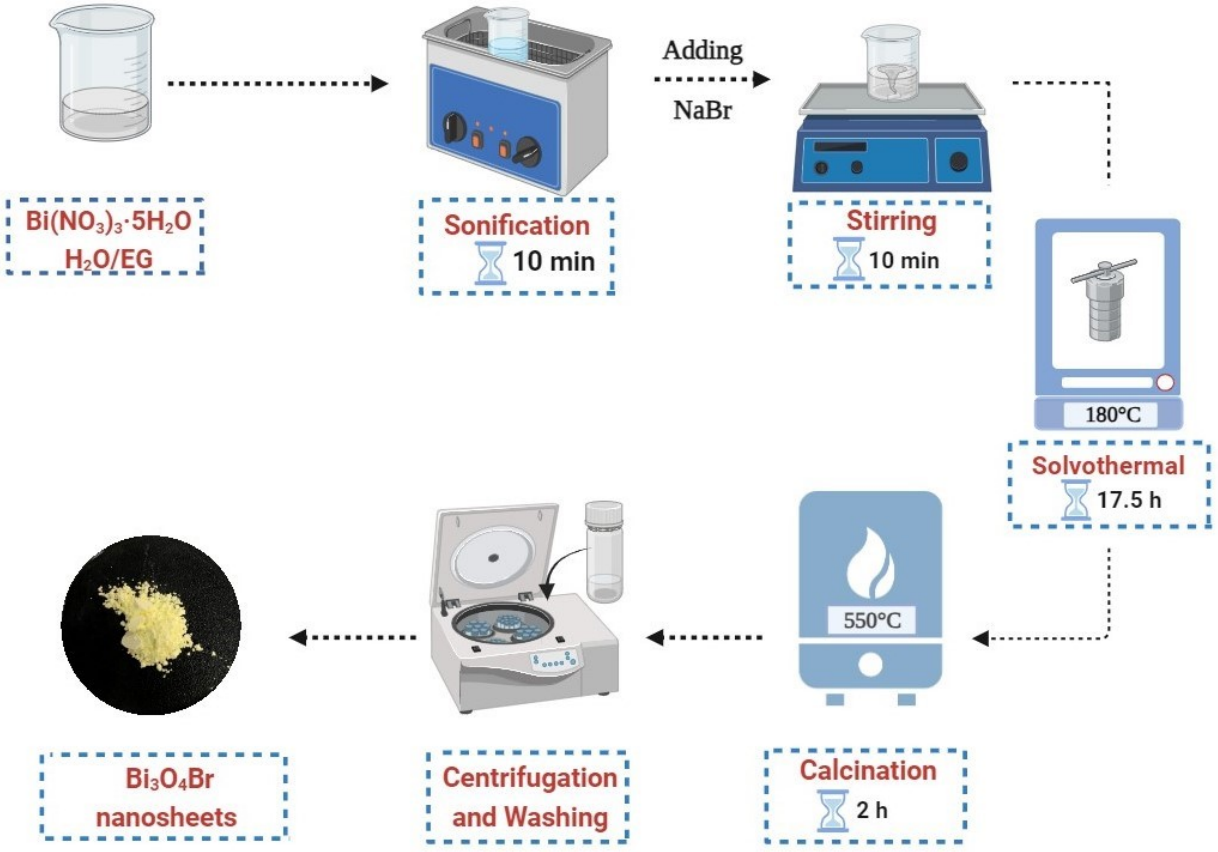

Figure S1 | Schematic illustration for the synthesis of Bi_3O_4Br nanosheets.

- **Preparation of Bi_3O_4Br @Chitosan Hybrid Materials**

Bi_3O_4Br@Chitosan hybrid materials were obtained by physically imprinting Bi_3O_4Br in chitosan beads through a co-precipitation method in a nonsolvent media. Briefly, 400 mg of chitosan and 150 mg of were sonically dispersed in 20 ml of H_2O for 1h. Then, 0,2 ml of acetic acid was added to the above solution under magnetic stirring to dissolve the chitosan. The formation of chitosan beads was achieved by dropping the obtained solution with a syringe in sodium hydroxide aqueous solution (200 mL, 2M), which causes the instantaneous precipitation of Bi_3O_4Br@Chitosan beads in the basic medium. After 1 hour in the alkaline solution, the Bi_3O_4Br@Chitosan beads were filtered and washed with distilled water several times until a neutral pH solution (pH=~7). A scheme of these synthetic steps is illustrated in Figure S2.

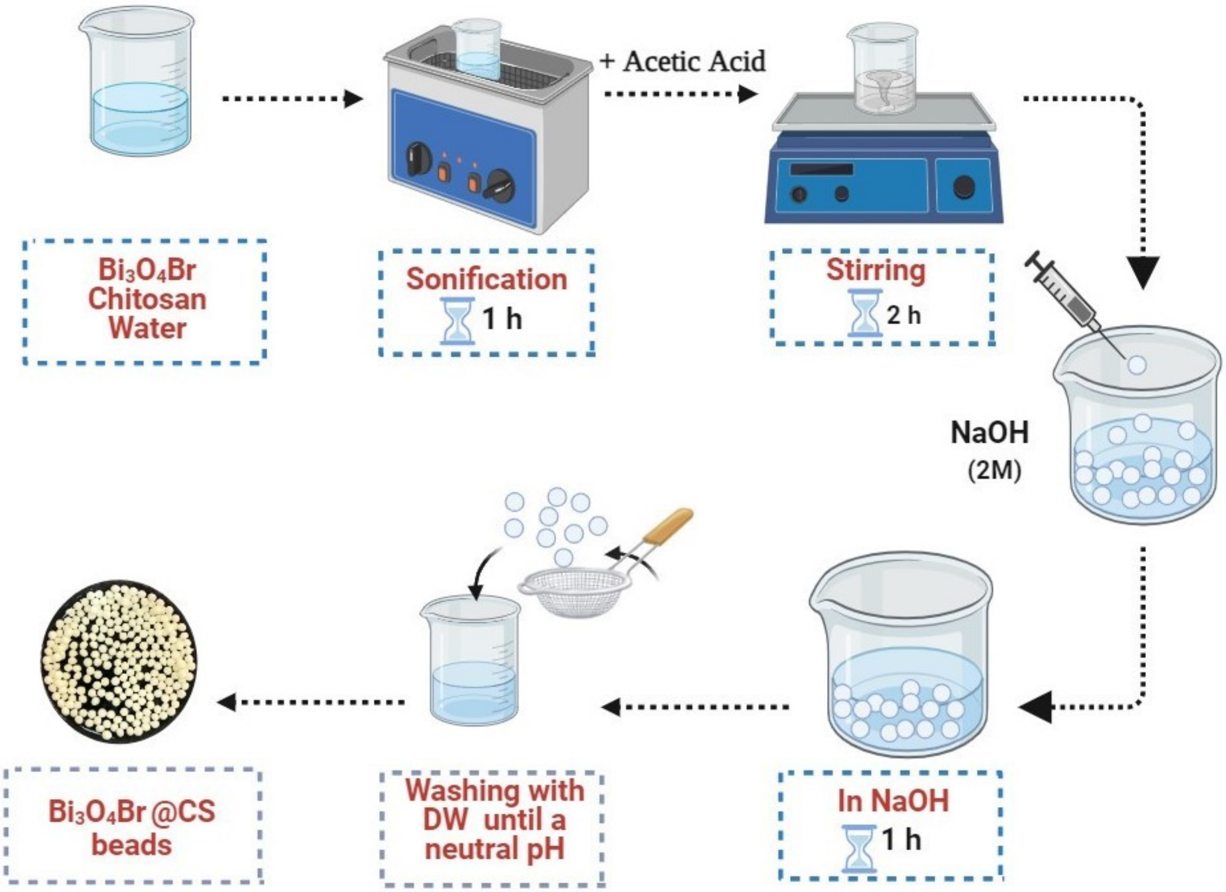

Figure S2 | Schematic illustration for the synthesis of Bi_3O_4Br@chitosan hybrid materials.

Catalytic and Photocatalytic Activity Evaluation

General Conditions for Rhodamine B (RhB) photodegradation

The photocatalytic behavior of the Bi_3O_4Br@CS hybrid materials was investigated in the photodegradation of organic dyes under UV light irradiation. For this, RhB was chosen as a model organic dye. These experiments were carried out in a quartz cuvette (25 mL) covered with a Teflon cover. In a typical photodegradation experiment, the quartz cuvette was charged with a certain amount of Bi_3O_4Br@CB and 6 mL of RhB aqueous solution. The reaction was stirred under simulated UV light using a Philips HPL-N 125W lamp with a wavelength of 365 nm as a light source. The progress of the photocatalytic reaction was performed by withdrawing a certain amount of RhB solution at regular intervals and monitoring the absorbance vs wavelength spectra using the UV-Vis spectrophotometer.

General Conditions for Reduction of 4-Nitrophenol to 4-aminophenol

The catalytic performance of Bi_3O_4Br@CS hybrid materials in the reduction of 4-NP was performed in an aqueous solution using $NaBH_4$ as a reducing agent. In a typical reduction process, a mixture of 6 mL of 4-NP aqueous solution (10 mg/L), Bi_3O_4Br@CSB and 2 mL NaBH4 (0.5, 1 or 2 M) was stirred at room temperature at selected period of time. The progress of the reduction process was determined by monitoring the absorbance band of 4-NP at 402 nm using UV-Vis spectrophotometer. At a regular time interval, samples of 4-NP solution were collected to plot the UV spectra of absorbance vs wavelength at each time.

TGA analysis

The thermal behavior of Bi_3O_4Br sheets and Bi_3O_4Br@CS composite has been studied by thermogravimetric analysis (TGA). The **figure S3** displays the TGA curves of Bi_3O_4Br sheets and Bi_3O_4Br@CS hybrid materials in the temperature range of (~20-700°C) with a heating rate of 10°C/min. The TGA curve of Bi_3O_4Br sheets revealed that no significant weight loss was observed in all temperature ranges, which confirms the thermal stability of the Bi_3O_4Br sheets in the range (20-700°C). Moreover, the absence of weight loss suggests that Bi_3O_4Br sheets have no residual organic compounds nor adsorbed water molecules. On the other hand, the TGA curve of Bi_3O_4Br @CS composite is composed of two regions separated by a significant weight loss in the temperature range of 200-300°C. This loss is mainly due to the degradation of chitosan polymeric structure above 250 °C. Above ~300°C, there is no obvious weight loss which indicates that chitosan has completely decomposed.

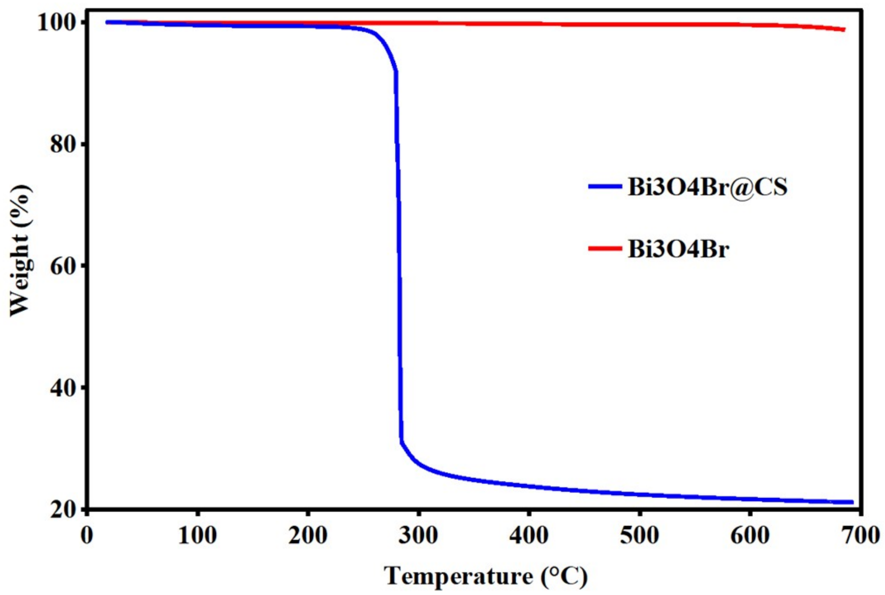

Figure S3 | TGA curves of Bi_3O_4Br sheets and Bi_3O_4Br @CS composite.

Additional SEM images

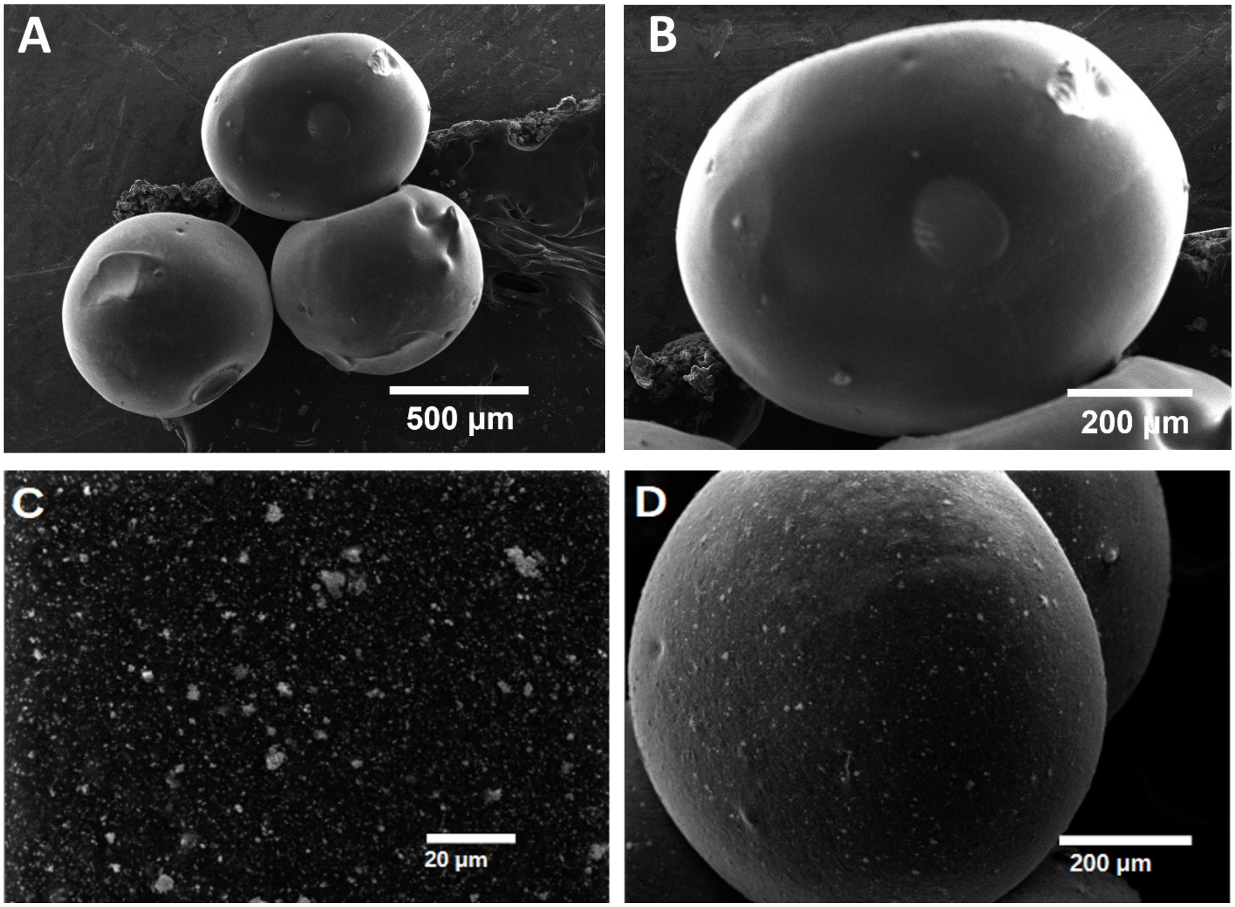

Figure S4 | SEM images of pure chitosan (A and B) and Bi_3O_4Br@Chitosan hybrid materials after three cycles (C and D).
